# Capturing relationships between suturing sub-skills to improve automatic suturing assessment

**DOI:** 10.1038/s41746-024-01143-3

**Published:** 2024-06-11

**Authors:** Zijun Cui, Runzhuo Ma, Cherine H. Yang, Anand Malpani, Timothy N. Chu, Ahmed Ghazi, John W. Davis, Brian J. Miles, Clayton Lau, Yan Liu, Andrew J. Hung

**Affiliations:** 1 University of Southern California, Los Angeles, CA USA; 2 Department of Urology, Cedars-Sinai Medical Center, Los Angeles, CA USA; 3 Surgical Science, Seattle, WA USA; 4 Johns Hopkins University, Baltimore, MD USA; 5 University of Texas MD Anderson Cancer Center, Houston, TX USA; 6 Houston Methodist, Houston, TX USA; 7 City of Hope, Duarte, CA USA

**Keywords:** Medical research, Health care

## Abstract

Suturing skill scores have demonstrated strong predictive capabilities for patient functional recovery. The suturing can be broken down into several substep components, including *needle repositioning*, *needle entry angle*, etc. Artificial intelligence (AI) systems have been explored to automate suturing skill scoring. Traditional approaches to skill assessment typically focus on evaluating individual sub-skills required for particular substeps in isolation. However, surgical procedures require the integration and coordination of multiple sub-skills to achieve successful outcomes. Significant associations among the technical sub-skill have been established by existing studies. In this paper, we propose a framework for joint skill assessment that takes into account the interconnected nature of sub-skills required in surgery. The prior known relationships among sub-skills are firstly identified. Our proposed AI system is then empowered by the prior known relationships to perform the suturing skill scoring for each sub-skill domain simultaneously. Our approach can effectively improve skill assessment performance through the prior known relationships among sub-skills. Through the proposed approach to joint skill assessment, we aspire to enhance the evaluation of surgical proficiency and ultimately improve patient outcomes in surgery.

## Introduction

Skill assessment is a fundamental aspect of surgical education and training, playing a pivotal role in evaluating the proficiency and competency of surgeons. Importantly, it has been demonstrated as a valuable and strong predictor for patient clinical outcomes^1–3^, underscoring its significance in ensuring optimal patient care. In a notable instance, manually assessed suturing technical skill scores emerged as the most robust predictors of patient continence recovery after a robot-assisted radical prostatectomy, outperforming other objective metrics of surgeon performance^4^. The need to automate skills assessment is evident, given that manual evaluations by expert raters are subjective, time-intensive, and lack scalability^5,6^. In recent years, advanced artificial intelligence (AI) systems have been leveraged to automate surgery technical skill scoring^7,8^, offering the potential for objective and efficient assessments. To enable a more precise evaluation of suturing technical skills, the process of suturing is usually deconstructed into substeps.

**Fig. 1:**
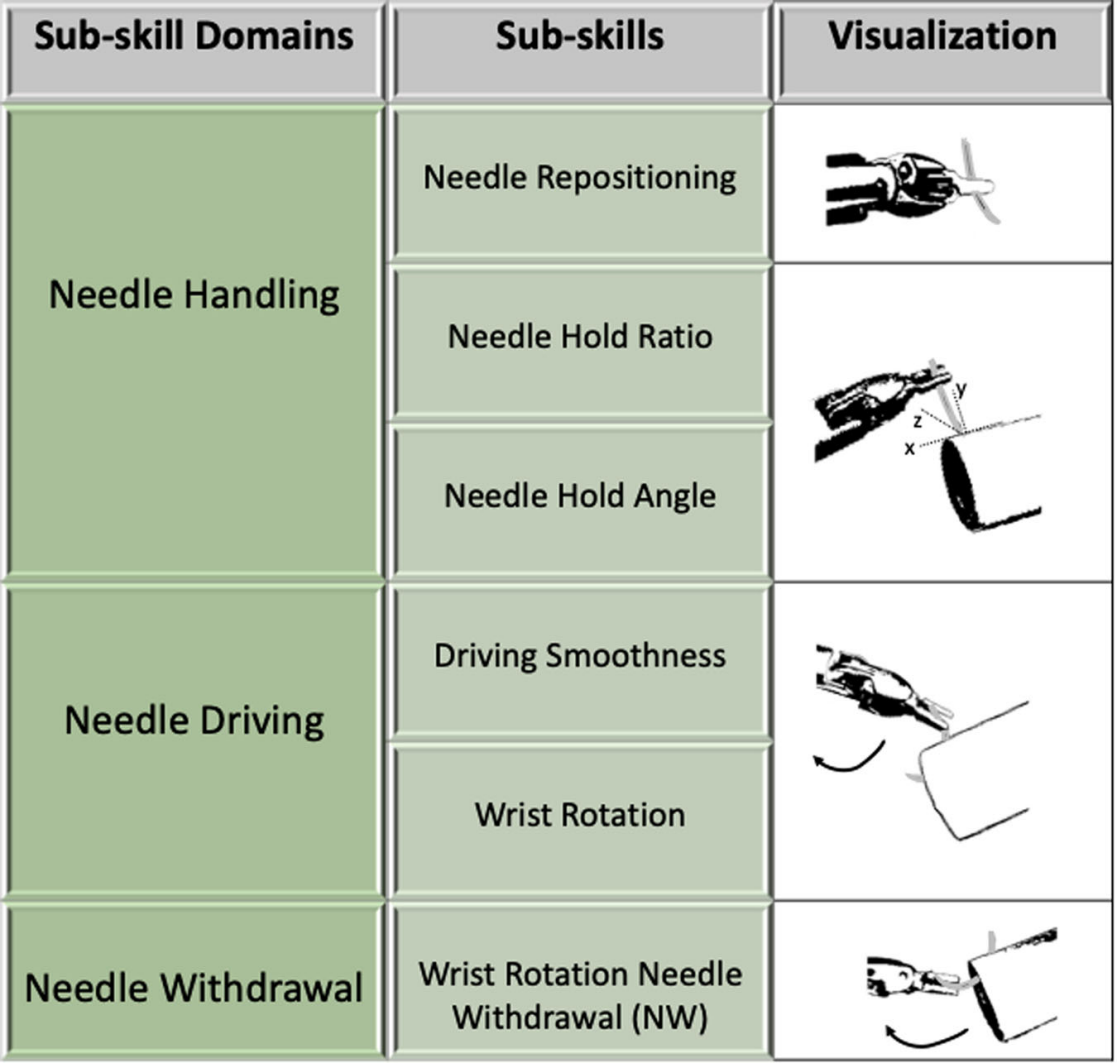
Sub-skill domains and sub-skills.

Considering three major sub-skill domains: needle handling, needle driving, and needle withdrawal, they are further divided into six sub-skills^9^. Needle handling involves three sub-skills: 1) *needle repositioning* represents how many times the needle exchange hands; 2) *needle hold ratio* represents if the holding is between 1/2 and 1/4 of a needle; 3) *needle hold angle* measures the needle angle with respect to tissue. Needle driving involves two sub-skills: 4) *driving smoothness* represents how smooth the needle drive in and out of tissue; 5) wrist rotation represents smooth wrist rotation during entry. Lastly, needle withdrawal involves one sub-skill: 6) *wrist rotation needle withdrawal (nw)* represents smooth wrist rotation during exit. We summarize sub-skill domains and sub-skills in Fig. 1. For existing systems, skill assessment has predominantly focused on the evaluation of individual sub-skills required for each substep in isolation. These methods provide valuable insights into the technical abilities of surgeons. However, the complex nature of surgical procedures necessitates a more comprehensive assessment approach that considers the integration and coordination of multiple sub-skills.

The successful outcomes of surgical procedures are contingent upon the integration and coordination of multiple sub-skills. Notably, existing study has revealed that technical skill scores between substeps of the suturing process are significantly associated^9^, further highlighting the relationships within the suturing workflow. For example, when *needle hold angle* is ideal, *needle withdrawal* has a significantly greater chance of being ideal. These findings emphasize the relationships among the technical sub-skills involved in completing various substeps of suturing. Consequently, machine learning efforts are expected to enhance automated skill assessment performance by harnessing these relationships and leveraging the power of data-driven approaches.

Examining the current deep learning techniques, graph neural networks (GNNs) have emerged as a powerful framework for capturing relationships in various domains, including social networks^10^, molecular chemistry^11,12^, recommendation systems^13^, and natural language processing^14^. Traditional neural networks (e.g., convolutional neural network^15^) are primarily designed for analyzing data with a grid-like structure, such as images^16^ or sequences^17^. They struggle to effectively model complex relationships among entities represented in graph structures. GNNs, on the other hand, excel at leveraging the inherent relationships present in graph data^18,19^. Given a graph structure, by propagating information through this structure, GNNs enable the modeling of hidden features based on their local neighborhoods. This unique ability to utilize relationships makes GNNs well-suited for tasks that require understanding complex relationships and interactions among entities. Lots of variants of GNNs are proposed, and among them, the graph attention network (GAT) has achieved a huge attention due to its embedded attention mechanism^20^. In this paper, we delve into the GAT and study its impact on utilizing relationships among surgical sub-skills for joint skill assessment. It is important to emphasize that our approach, while falling under the umbrella of multi-task learning, distinguishes itself from existing developments on the public JIGSAWS dataset^21^. Those multi-task pipelines primarily consider the recognition of synchronized surgical gestures^22,23^ as the additional task. To the best of our knowledge, our work represents a pioneering effort in multi-task learning by considering the intricate relationships that exist among surgical sub-skills.

**Fig. 2:**
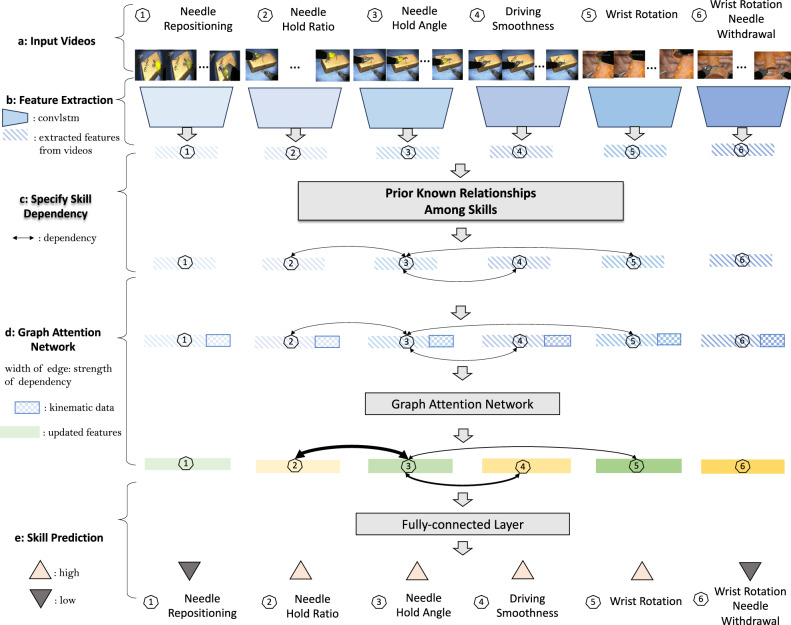
Overview of the joint skill assessment. **a** Input videos clips for each sub-skills that are within a natural suturing. **b** Extraction of isolated features for each sub-skill from videos. There is no relationship among sub-skills during feature extraction. **c** Incorporation of prior known relationships, that indicate the association among sub-skills. **d** Graph attention network given specified prior known relationships among sub-skills. Attention mechanism automatically adjusts the strength of the association among sub-skills. Kinematic information is introduced as additional input for the graph attention network to further enhance the joint skill assessment. **e** Final skill assessment given the updated features from the graph attention network.

In this study, we propose a framework for joint skill assessment that takes into account the interconnected nature of sub-skills required in surgery. Our approach can be applied to any individual skill assessment baselines. Given the known relationships between sub-skills as our prior, we embed them as a graph structure and leverage the GAT for joint skill assessment. Given the fact that the strength of associations can vary, the GAT helps automatically adjust the strength of associations through its attention mechanism. When available, kinematic information is introduced for joint skill assessment to help refine the strength of associations and enhance the performance. The overview of the proposed method is shown in Fig. 2. We evaluated the proposed framework using virtual reality (VR) videos collected from a VR simulator, specifically focusing on the surgical procedure of robot-assisted radical prostatectomy. Through evaluation, the proposed framework, serving as a surgical education assessment tool, is effective for surgical education and training.

## Results

### Prior known relationships among sub-skills

We firstly define the prior known relationships among sub-skills. In Fig. 2 (Part c), we visualize the prior known relationships among sub-skills. Solid links represent the relationships. As shown, the associations between sub-skills are reasonably well aligned with our prior work demonstrating the inter-skill relationships within suturing^9^. For example, *wrist rotation needle withdrawal* is associated with *hold angle* directly.

### Joint skill assessment helps improve skill assessment performance

We study the effectiveness of relationships among sub-skills in improving skill assessment performance. We leverage the known relationships (i.e., the edges in Fig. 2 Part c) as our prior to define the association among nodes. In particular, the prior known relationships consists of three pairs: *hold ratio* and *hold angle*, *hold angle* and *driving sequence*, *hold angle* and *wrist rotation*.

We compare the skill assessment performance for six different sub-skills before and after performing the joint skill assessment. We report the average assessment accuracy for each skill over five different institutions in Table 1. As shown, on average over six different sub-skills, the assessment performance improves by leveraging the relationships. Most of the sub-skills achieve improved assessment accuracy by leveraging the prior known relationships. For example, *needle hold angle* achieves 6% improvements because of informative information collected from its neighboring sub-skills (i.e., *needle hold ratio*, *driving smoothness*, and *wrist rotation*) through joint skill assessment.

**Table 1:** Compare joint skill assessment performance to independent skill assessment performance

Sub-skill	Independent	Joint (Ours)
Needle Repositioning	0.72 ± 0.09	**0.80** ± **0.03**
Needle Hold Ratio	0.57 ± 0.04	**0.60** ± **0.03**
Needle Hold Angle	0.53 ± 0.05	**0.59** ± **0.04**
Needle Driving Smoothness	**0.87** ± **0.05**	0.86 ± 0.04
Wrist Rotation	0.57 ± 0.05	**0.64** ± **0.06**
Wrist Rotation Needle Withdrawal	0.64 ± 0.07	**0.69** ± **0.04**
**MEAN**	0.65 ± 0.02	**0.70** ± **0.02**

Boldfaced denotes best and ± are standard deviations across 5 held-out institutions (AUC).

**Fig. 3:**
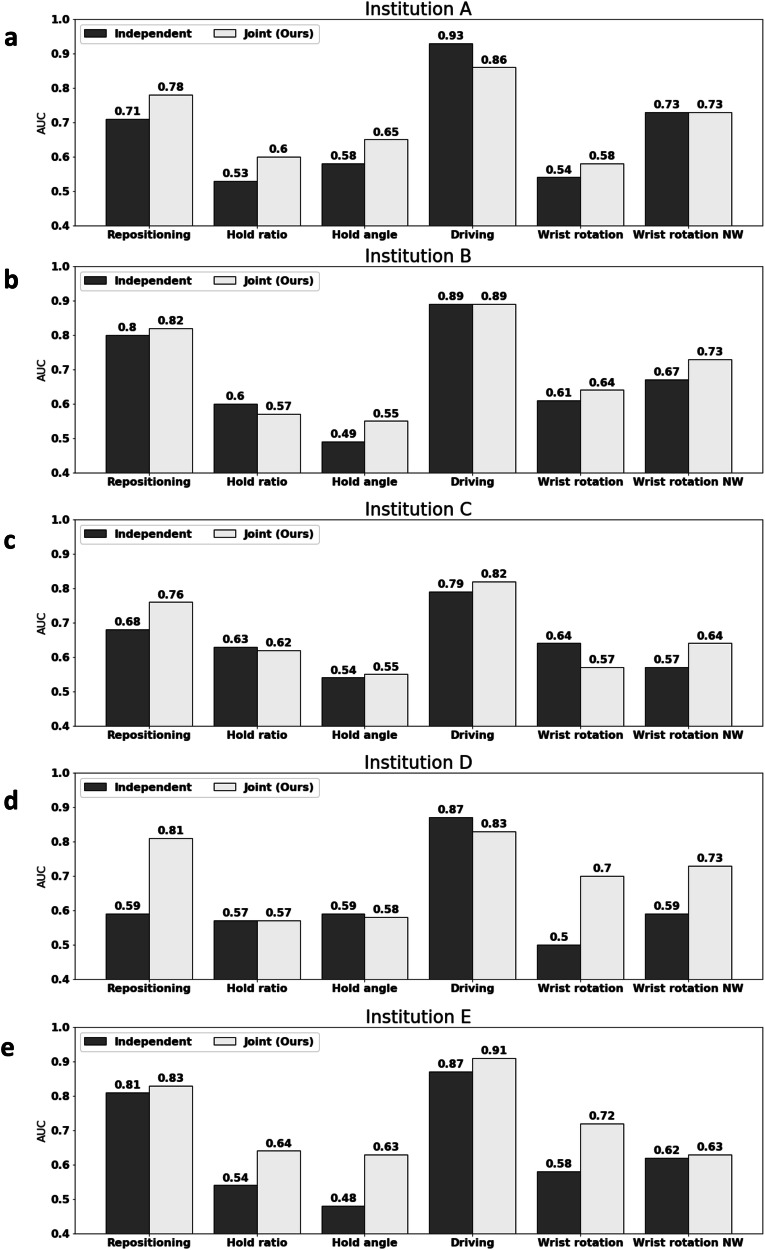
Comparing joint skill assessment performance to independent skill assessment performance at each institution. **a**–**e** Performance at Institution A–E. The *X*-axis represents six different sub-skills. The *Y*-axis represents the AUC score.

Furthermore, for each of the 5 institutions that have contributed data, we visualize the skill scoring for each sub-skill. As shown in Fig. 3, joint skill assessment helps improve performance across different institutions. *Needle hold angle* and *wrist rotation* achieve performance improvements at most of the institutions. For example, *wrist rotation* improves 19.6% and 13.5% at institution D and E, respectively.

### Learnable attention weights in GAT help adjust association strength

Though we define the prior known relationships among sub-skills, the association strength among sub-skills could vary. The learnable attention mechanism allows for adjustable weights to better perform the joint skill assessment. For comparison, we consider the baseline model without any learnable attention mechanism. The strength of the relationships is fixed to one and doesn’t vary. We report the average skill assessment performance across five institutions in Table 2.

**Table 2:** Effectiveness of learnable attention weights in GAT

Sub-skill	w/o Attention	w Attention
Needle Repositioning	0.79 ± 0.06	**0.80** ± **0.03**
Needle Hold Ratio	0.60 ± 0.05	0.60 ± 0.03
Needle Hold Angle	0.54 ± 0.06	**0.59** ± **0.04**
Needle Driving Smoothness	0.83 ± 0.07	**0.86** ± **0.04**
Wrist Rotation	0.62 ± 0.07	**0.64** ± **0.06**
Wrist Rotation Needle Withdrawal	**0.70** ± **0.04**	0.69 ± 0.04
**MEAN**	0.68 ± 0.05	**0.70** ± **0.02**

Boldfaced denotes best and ± are standard deviations across 5 held-out institutions (AUC).

As shown, adjusting the strength of association through learnable attention mechanism help perform effective skill assessment. Most of the sub-skills achieve improved performance through adjustable association strength. For example, *needle hold angle* and *needle driving smoothness* improve performance compared to the one without leveraging attention mechanism. Though we leverage the prior known relationships among sub-skills, the strength of these relationships can vary across different institutions, hence adopting the attention mechanism for adjustable association strength helps.

### Relationships generalize across different institutions

**Fig. 4:**
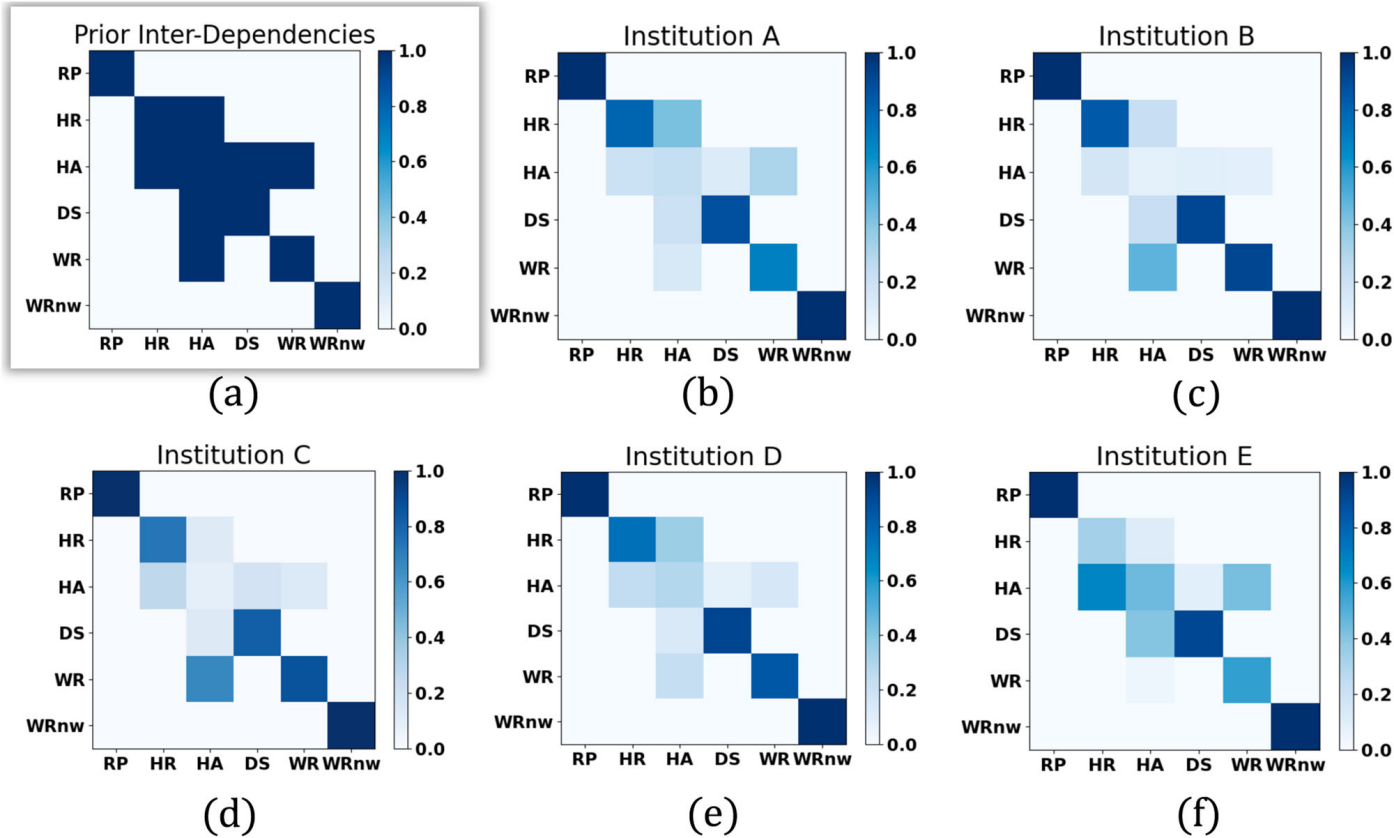
Visualization of relationships among sub-skills. Each row (and column) represents one sub-skill. For clarity, we use abbreviation RP, HR, HA, DS, WR, WRnw to respectively index six sub-skills: *needle repositioning*, *needle hold ratio*, *needle hold angle*, *needle driving smoothness*, *wrist rotation*, *wrist rotation needle withdrawal*. The value in *i*-th row and *j*-th column represents the association from *i*-th sub-skill to *j*-th sub-skill. **a** Shows the prior known relationships among six sub-skills, whose elements are of value one or zero, indicating the existence of an edge between two nodes or not. **b**–**f** The attention map among sub-skills for each institution. Each element of the attention map is of value within the range of [0,1]. The deeper the blue, the stronger the association.

To further study the effectiveness of the relationships across different institutions, for each institution, we visualize the attentions among different sub-skills in Fig. 4. For each sub-skill, it can only pay attention to its neighboring sub-skills defined by the prior relationships and itself. The off-diagonal values in the attention map represent the associations between each sub-skill and its neighboring sub-skills, while the diagonal values denote the association of each sub-skill with itself. The attention values vary and are learned during training. It is worth noting that the diagonal attention values may not always surpass the off-diagonal values. In the case of the sub-skill *needle hold angle*, its weak self-association implies that the information from its neighboring sub-skills (i.e., *needle wrist rotation*) holds more weight in skill assessment than its own information. For comparison, we visualize the prior relationships among sub-skills in Fig. 4a.

Interestingly, we observe some common patterns in attention maps that are shared across different institutions. The diagonal values are usually stronger than the off-diagonal values, particularly for sub-skill 2 (*needle hold ratio*), sub-skill 4 (*needle driving sequence*) and sub-skill 5 (*wrist rotation*). For sub-skill 3 (*needle hold angle*), it is likely to have stronger association with its neighboring sub-skills than itself. For example, at institution A and D, the association between sub-skill 3 (*needle hold angle*) and sub-skill 2 (*needle hold ratio*) is stronger. At institutions B and C, the association between sub-skill 3 (*needle hold angle*) and sub-skill 5 (*wrist rotation*) is stronger. Besides, the off-diagonal values are almost symmetric, particularly for the pair sub-skill 3 (*needle hold angle*) and sub-skill 4 (*needle driving smoothness*).

### Kinematics helps joint skill assessment

We study the effectiveness of the kinematic (i.e., the instrument motion-tracking data) in joint skill assessment. The prior known relationships define the association among sub-skills. Besides, kinematic data is introduced to help refine the association strength and further enhance skill assessment performance. We compare the performance of joint skill assessment with videos only and with videos plus kinematic data. We report the average skill assessment performance across five institutions in Table 3.

**Table 3:** Effectiveness of kinematics in joint skill assessment

Sub-skill	Videos	Videos + Kim.
Needle Repositioning	0.74 ± 0.07	**0.80** ± **0.03**
Needle Hold Ratio	0.59 ± 0.04	**0.60** ± 0.03
Needle Hold Angle	0.52 ± 0.06	**0.59** ± **0.04**
Needle Driving Smoothness	**0.88** ± 0.04	0.86 ± 0.04
Wrist Rotation	0.61 ± 0.07	**0.64** ± **0.06**
Wrist Rotation Needle Withdrawal	0.66 ± 0.05	**0.69** ± **0.04**
**MEAN**	0.67 ± 0.02	**0.70** ± **0.02**

Boldfaced denotes best and ± are standard deviations across 5 held-out institutions (AUC).

As shown, performing joint skill assessment with kinematics leads to significantly improved performance. For most of the sub-skills, the performance is improved with kinematics. For example, both *wrist rotation* and *wrist rotation needle withdrawal* improves 3%. Besides, it is also worth noting that, without kinematics, the joint skill assessment still achieves performance improvement on average compared to the independent skill assessment (0.67 versus 0.65).

### Effectiveness in improving patient outcomes

To compare the effectiveness of our proposed automated suturing skills assessment approach against human ratings in predicting postoperative outcomes, we have expanded our analysis to include 3-month patient continence recovery after robot-assisted radical prostatectomy (RARP). All surgeons who contributed VR suturing skill demonstration also contributed functional recovery data for their patients after RARP. We first examine the association between EASE skill scores (generated by our model or human raters) and the 3-month continence recovery. The results are displayed in Tables 4 and 5, respectively. As shown, our automated EASE scores demonstrate significant association with 3-month continence recovery across three skill domains (*P*-value < 0.05), whereas human-generated EASE scores exhibit significant association in only one domain. This disparity underscores the strengthened association of our model with 3-month continence recovery and highlights its potential to inform surgical training strategies and improve patient outcomes.

**Table 4:** Association between automated EASE scores generated by our model (denoted as “AI") and the 3-month continence recovery

AI	Rate ratio	95% Confidence Interval	*p* value
RP	1.868	1.249–2.793	0.002
HR	0.555	0.301–1.022	0.059
HA	0.207	0.037–1.166	0.074
DS	0.538	0.334–0.868	0.011
WR	3.986	1.007–15.782	0.049
WRnw	1.885	1.158–3.067	0.011

For clarity, we use abbreviation RP, HR, HA, DS, WR, WRnw to respectively index six sub-skills: needle repositioning, needle hold ratio, needle hold angle, needle driving smoothness, wrist rotation, wrist rotation needle withdrawal.

**Table 5:** Association between EASE scores generated by human raters (denoted as “Human”) and the 3-month continence recovery

Human	Rate Ratio	95% Confidence Interval	*p* value
RP	1.933	1.288–2.902	0.001
HR	1.032	0.648–1.642	0.895
HA	0.761	0.532–1.087	0.133
DS	0.821	0.257–2.62	0.739
WR	1.617	0.954–2.741	0.074
WRnw	0.827	0.546–1.253	0.37

For clarity, we use abbreviation RP, HR, HA, DS, WR, WRnw to respectively index six sub-skills: needle repositioning, needle hold ratio, needle hold angle, needle driving smoothness, wrist rotation, wrist rotation needle withdrawal.

**Fig. 5:**
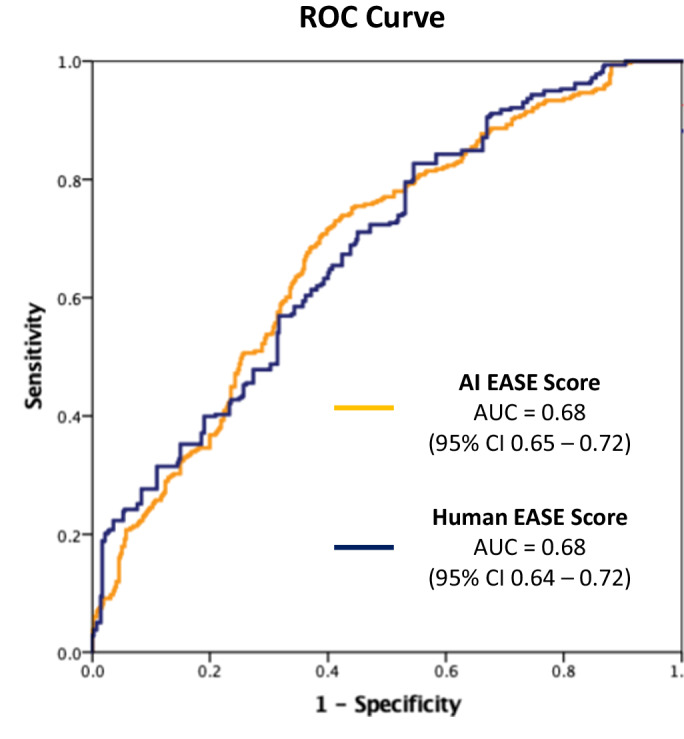
The predictive accuracy of EASE scores for 3-month continence recovery. AI EASE scores represent the EASE scores generated by our proposed model, and human EASE scores represent the EASE scores from human raters.

We further assess the predictive accuracy of EASE scores (from our model or human raters) for 3-month continence recovery. The ROC curve is plotted in Fig. 5. Our analysis reveals comparable performance between our proposed model and the human ratings, both achieving an AUC of 0.68 for predicting 3-month continence recovery, with patient features adjusted (i.e., age, BMI, PSA, and prostate volume). This equivalence in performance underscores the effectiveness of our proposed model in predicting 3-month continence recovery, aligning closely with the predictive capabilities of experienced human evaluators.

## Discussions

Skill assessment is an essential part of surgery to identify the competence of the surgeons. It has important implications for surgical training, surgeon accreditation, and patient outcomes. However, manual suturing skill assessments carried out by experienced surgeons require a deep understanding acquired through years of practice, and laborious process prone to observer biases. With the development of AI systems in recent years, there is a need to automate the process of skills assessment by levaraging the learning power of AI through data. Several papers previously examined different models to perform skills assessment tasks and established various baseline models. The prior research work on this topic evaluated the skills independently. With well-established relationships between sub phases of suturing, we explore the possibility of incorporating such prior known relationships for joint skill assessment through the graph attention network.

To perform joint skill assessments, we perform graph convolution on top of the individual sub-skill assessment. We perform 5-fold cross-validation given data collected from five institutions. We observe that joint skill assessment helps improve the performance consistently compared to individual skill assessment performance across different institutions. Notably, our findings also reveal commonalities in the strength of associations shared among different institutions. Numerous studies have been undertaken to evaluate the effectiveness of each component within the proposed model. A summary of these findings is provided in the Table 6, offering overall insights into the contribution of individual elements to the overall performance. Additionally, calibration plots for each sub-skill are appended (Supplementary Fig. 1), offering a visual depiction of calibration performance and further enhancing the comprehensiveness of the analysis.

**Table 6:** Summary of results

Sub-skill	Independent	Joint		
		w/o Attention	w/o Kinematics	Ours
Needle Repositioning	0.72 ± 0.09	0.79 ± 0.06	0.74 ± 0.07	**0.80** ± **0.03**
Needle Hold Ratio	0.57 ± 0.04	0.60 ± 0.05	0.59 ± 0.04	**0.60** ± 0.03
Needle Hold Angle	0.53 ± 0.05	0.54 ± 0.06	0.52 ± 0.06	**0.59** ± **0.04**
Needle Driving Smoothness	0.87 ± 0.05	0.83 ± 0.07	**0.88** ± **0.04**	0.86 ± 0.04
Wrist Rotation	0.57 ± 0.05	0.62 ± 0.07	0.61 ± 0.07	**0.64** ± **0.06**
Wrist Rotation Needle Withdrawal	0.64 ± 0.07	**0.70** ± **0.04**	0.66 ± 0.05	0.69 ± 0.04
**MEAN**	0.65 ± 0.02	0.68 ± 0.05	0.67 ± 0.02	**0.70** ± **0.02**

Boldfaced denotes best and ± are standard deviations across 5 held-out institutions (AUC).

Although joint skill assessment shows improvement compared to individual assessments, its performance is notably affected by the strength of associations. This sensitivity can be attributed to the attention weights used in the joint skill assessment. We hypothesize that the strength of associations varies across different institutions. Hence, employing a learnable attention mechanism can help enhance the effectiveness of joint skill assessment. Our results validate our hypothesis by showing that an attention mechanism contributes to an average performance improvement of 2%, in comparison to a scenario where attention weights are fixed at one (Table 2). This demonstrates the significance of adapting attention weights through learning and underscore the pivotal role of associations strength in joint skill assessment. Notably, the significance of attention mechanism depends on its complexity and its inputs. In our work, it is a shallow neural network, only employed for adjusting the associations strength among six sub-skill. Thus, its effectiveness may not as compelling as the ones employed for temporal modeling^8^.

Additionally, we observe that *driving smoothness* doesn’t exhibit a substantial performance improvement through joint skill assessment. This can be attributed to the fact that the individual assessment of *driving smoothness* already achieves a remarkable performance with AUC 87%. Through our investigations, we observe that joint skill assessment demonstrates heightened effectiveness particularly for sub-skills that can not be robustly assessed in isolation.

The major limitation of the present work is rooted in the reliance of joint skill assessment on kinematic data to achieve notable performance gains. As shown, when kinematic data is omitted, the average enhancement achieved by joint skill assessment is 2% compared to the individual skill assessment. While when kinematic data is harnessed, the average improvement escalates to 5%. From these studies, we observe that the efficacy of joint skill assessment hinges on the nature of input information. Besides, the prior known relationships among sub-skills are determined offline and remain fixed throughout training. This can potentially be a limitation as the relationships among sub-skills are assumed to be same across different institutions.

Another limitation of our study pertains to the medical insights derived, which are specific to the urological procedure under examination. While we delved into the correlation with urinary continence outcomes in urology, this association remains confined within the scope of urological practice. However, from a technical standpoint, the deep learning pipeline proposed in our study exhibits inherent adaptability to various procedures and is not confined to any particular virtual platform; it can apply to platforms like da Vinci. Furthermore, our VR exercise concentrated on tube suturing, a skill agnostic to any surgical specialty, indicating its broad applicability. The suturing domains under consideration—needle handling, needle driving, and needle withdrawal—are also generalizable across surgical disciplines.

Our future work will involve delving into an end-to-end joint skill assessment approach that encompasses feature extraction. Additionally, we intend to explore a mechanism for a learnable structure, allowing for adaptable relationships among sub-skills. By addressing these aspects, we aim to enhance the versatility and robustness of joint skill assessment, advancing its applicability in diverse surgical contexts. Our work considers the virtual reality (VR) videos. VR videos offer a safe and repeatable platform for a comprehensive study in a controlled and lifelike setting. Moreover, they bear similarities to live videos in terms of the complex dynamics and reasonable visual details. The insights of the proposed joint skill assessment pipeline learned from VR videos offer promising avenues for advancements in live surgical video analysis and training in the future.

Finally, it is essential to highlight an open-ended question regarding the use of human ratings of suturing skills as the gold standard. While the ultimate goal remains to improve patient outcomes, our past research found that in the real world, there are many confounding factors for patient outcomes, including the most apparently, patient features. These factors, often beyond the control of surgeons, pose challenges in accurately attributing outcomes solely to surgical technique. Indeed, distinguishing the proportion of variability attributable to surgeon technique is a technically challenging task. Looking ahead, we would like to conduct comprehensive studies to unravel this complex interplay, aiming to elucidate the distinct contributions of surgical technique and patient characteristics toward achieving optimal patient outcomes.

## Methods

### Ethics approval

All datasets used in this study were collected following rigorous ethical standards under the approval of the Institutional Review Board (IRB) of the University of Southern California, ensuring the protection of participants’ rights and privacy. Written informed consent was obtained from all individuals who participated in the dataset collection (HS-17-00113). Furthermore, to safeguard the privacy and confidentiality of the participants, the datasets were de-identified prior to model development or analysis.

### Description of surgical procedure and activities

This study focused on robot-assisted radical prostatectomy (RARP). This is a surgical procedure utilized for the treatment of prostate cancer. The essence of this surgical procedure lies in the meticulous execution of sequential steps by the surgeon. Vesicoureteral anastomosis (VUA) is one specific step of the RARP procedure. During the VUA step, a reconstructive suturing process takes place, where the bladder and urethra, previously separated by the removal of the prostate, are stitched together. This connection is vital to facilitate the normal flow of urine postoperatively. The suturing process in the VUA step involves three essential actions: needle handling, wherein the surgeon grasps the needle with one of the robotic arms; needle driving, which entails pushing the needle through the tissue; and needle withdrawal, wherein the needle is withdrawn on the other side of the tissue in preparation for the subsequent stitch.

### Surgical video samples and annotations

We utilize a previously validated suturing assessment tool (EASE^9^) to evaluate the proposed joint skill assessment framework.

**Fig. 6:**
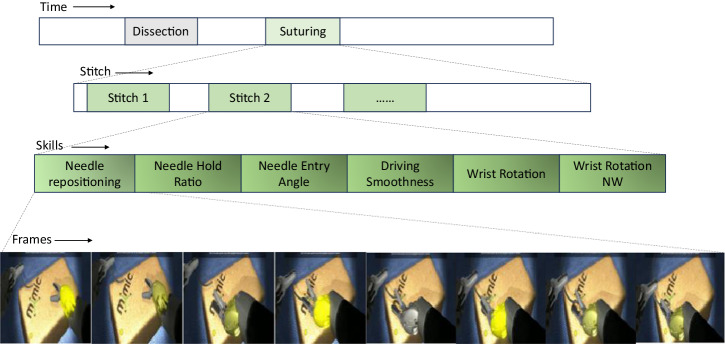
A suturing video is firstly divided into individual stitches. Each stitch is further segmented into sub-phases, corresponding to six surgical sub-skills.

We collected a total of 156 virtual reality (VR) videos from 43 residents, fellows, and attending urologic surgeons in a 5-center multi-institutional study. VR suturing exercises were completed on the Surgical Science™ Flex VR simulator. Each video was further divided into individual stitches, totally 3448 stitches. An illustrative visualization is shown in Fig. 6. Moreover, each stitch was segmented into sub-phases, encompassing six binary assessment labels for technical sub-skill (low vs. high skill). Six independent and blinded raters underwent standardized training, which involved 2-hour prerecorded video sessions and 10 VR scoring practice exercises at the stitch level, utilizing the EASE-VR scoring system. After training, they conducted assessments of technical skills in VR exercises utilizing EASE. Inter-rater reliability was quantified using the prevalence-adjusted bias-adjusted kappa (PABAK) statistic. The evaluators attained a median PABAK of 0.74 (interquartile range [IQR] 0.62–0.86), across six skill domains.

For patient outcome, robotic-assisted radical prostatectomy cases of participating surgeons from 5 institutions were included. All surgeons who contributed VR suturing skill demonstration also contributed functional recovery data for their patients after RARP. Patient data was obtained by chart review, including age, BMI, PSA, and prostate volume. Follow-up data at 3 months were obtained by chart review or telephone by an independent research coordinator utilizing patient-reported outcomes. Urinary continence recovery is defined as 0 or 1 safety pad use per day.

Videos are segmented into sub-phases based on labeled timestamps. Standard preprocessing techniques for video data, including channel normalization and center cropping of the spatial dimensions to 224 × 224 pixels, were applied. Kinematic data has 70 features tracking 10 points of interest, each pose contains 3 elements for coordinates and 4 elements for quarternions. Particularly, 10 points of interest include two needles, left and right pose, left and right target pose, insertion pose, and extraction pose. To synchronize kinematics data, we extract frame ids from each clip and unitize them to align kinematics frame-by-frame from raw sensor logs. For kinematics, all measurements are firstly centered by subtracting the camera position and multiplying by the camera’s inversed orientation, then normalized by the maximum distance from the origin.

### Joint skill assessment framework

We propose a joint skill assessment framework which considers the prior known relationships among sub-skills.

Our approach consists of two stages. For the first stage, we extract features for each individual features using existing approaches. The extracted features for each sub-skill will serve as inputs for the second stage. The second stage performs graph convolution. After performing the graph convolution, the second stage outputs the updated features for each node (sub-skill). The final assessment is performed given the updated features for each sub-skill. The input for a suturing sub-skill consisting of segmented video clips and aligned kinematic sequences, and EASE technical sub-skill score for non-ideal vs ideal performance.

*Stage 1:* In Stage 1, we consider the Convolutional Long Short-Term Memory (ConvLSTM) as our baseline model for individual sub-skill assessment. ConvLSTM is a specialized neural network architecture that combines the strengths of convolutional neural networks (CNNs)^24^ and Long Short-Term Memory (LSTM) cells^25^. ConvLSTM is widely used in video understanding tasks, due to its ability to capture both spatial and temporal information. Building on prior research, we not only consider the visual appearance of videos but also incorporate the optical flow between two consecutive frames to enhance the individual sub-skill assessment process.

*Stage 2:* In Stage 2, we consider the Graph Attention (GAT) module^26^ for joint skill assessment. We represent each individual sub-skill as a node in a graph. The association among nodes in the graph is determined based on the prior known relationships between different sub-skills in the suturing process. The extracted features from Stage 1 are firstly concatenated to the aligned kinematic data for each sub-skill. The concatenated features serve as input features for each node in GAT. GAT performs graph convolution. Given the input features and the kinematic data for each node (i.e., sub-skill) and the association among nodes, GAT updates the features of each node (representing a sub-skill) by aggregating information from its neighboring nodes. The association among nodes specifies which sub-skills are considered as neighbors and will be used to collect relevant information for each node’s update.

Different from other graph convolution approaches, an attention mechanism is employed in GAT. It enables GAT to automatically adjust the edge weights and to focus on more informative and relevant neighbors during the feature aggregation step. This attention mechanism assigns different attention weights to the neighboring nodes, allowing the model to emphasize the most relevant information and effectively capture the complex dependencies among different sub-skills.

Encompassing data from five institutions, we adopt 5-fold cross-validation for training and evaluation. We iteratively train our model on data from four institutions while evaluate its performance on the fifth held-out institution. We repeat this process five times to ensure that each institution’s data is used for both training and testing. This cross-validation strategy not only accounts for variations across individual surgeons but also allows us to test the model’s ability to generalize to unseen cases across multiple medical centers.

During the training process, we implement a two-stage straining strategy. In Stage 1, we train a baseline model for each sub-skill independently. The primary objective is to optimize the performance of each baseline model, and the training loss is computed using cross-entropy, which measures the discrepancy between the predicted sub-skill assessments and the ground truth labels for each sub-skill. Once Stage 1 training is completed, we collect distinct features for each sub-skill from its respective trained baseline model. In Stage 2, we train the joint skill assessment model, utilizing the collected features from Stage 1 and the aligned kinematic data. The primary objective is to optimize joint skill assessment in an integrated manner. The training loss is the summation of the cross-entropy, calculated between the six predicted sub-skill assessments and the corresponding ground truth sub-skill labels. The training of Stage 2 is independent of the training conducted in Stage 1. This two-stage training strategy enables us to refine the skill assessment by firstly focusing on individual sub-skill models and then leverage the prior known relationships to perform a robust joint skill assessment.

### Skill assessment evaluation metric

In our evaluation process, we measure and report the mean ± standard deviation (std. dev.) for the Area-under-the-ROC curve (AUC) metric across the five test folds. AUC is a widely used performance metric in binary classification tasks. The ROC (Receiver Operating Characteristic) curve is a graphical representation that illustrates the trade-off between the true positive rate (or sensitivity) and the false positive rate (or 1 − specificity) at various classification thresholds. The AUC ranges from 0 to 1 and quantifies the overall discriminatory power of the model. A higher AUC indicates better classification performance. We use the AUC metric in our study to evaluate the performance of our model in distinguishing between high-skill and low-skill. By calculating the mean AUC, we obtain the average predictive capability of the model across different test folds. Additionally, the std. dev. reflects the variability of AUC scores, indicating the robustness of the model’s performance on unseen samples from different medical centers.

### Implementation details

In Stage 1, we use the ConvLSTM, which uses a pre-trained AlexNet to extract visual and flow features from the penultimate layer for each frame. The features are then flattened as used as input vectors to the sequential model ConvLSTM. The output feature dimension is 128. In Stage 2, we first concatenate the feature from Stage 1 to the kinematic data sequence. For each sub-skill, the sequence length is 24 and the kinematic data for each frame has 70 features. In total, for each sub-skill (i.e., node), the input dimension is 128 + 70 * 24 = 1808. The hidden dimension is 1024 and the output dimension is 128. For attention mechanism, the hidden dimension is 1024 with one layer. The learning rate is 1e−3 and the total number of epoch is 50.

### Association between suturing skills and clinical outcomes

The association between suturing skill domains and surgeons’ clinical outcome (i.e., recovery of continence at 3 months,) was calculated by univariable logistic regression. Then a multivariable logistic regression model was created to adjust for patient features, comprising of age, BMI, PSA, and prostate volume. All VR suturing skill domains were included in this multivariable model as well. The predicted value for each patient in the multivariable model was used to create receiver operating characteristic (ROC) curve, and area-under-the-curve (AUC) was calculated. This process was conducted for AI-generated and human-generated VR suturing skill scores separately. AUCs were compared between AI and human.

### Supplementary information

supplemental material

## Data Availability

The datasets generated during and/or analyzed during the current study are available from the corresponding author on reasonable request.

## Code availability

The codes used in this study is available from the corresponding author upon reasonable request.
