# Supplementary material for: Capturing relationships between suturing sub-skills to improve automatic suturing assessment
Source: NPJ Digit Med. 2024 Jun 11;7:152. doi: 10.1038/s41746-024-01143-3 (PMC11167055; doi:10.1038/s41746-024-01143-3)
Supplement: Supplementary file 1 — supplemental material [file 41746_2024_1143_MOESM1_ESM.pdf]

Supplemental Materials

Supplemental Discussion

Calibration plots provide a graphical representation of the relationship between predicted probabilities and observed outcomes, offering valuable insights into the calibration performance of predictive models. The ideal scenario is for the points to align closely along a diagonal line. Calibration plots for each sub-skill are shown in Figure 1. In these plots, the x-axis represents the predicted probabilities, while the y-axis depicts the observed outcomes. By examining calibration plots for each sub-skill, we can assess the model's reliability and accuracy across different sub-skills, thereby gaining a deeper understanding of its predictive performance.

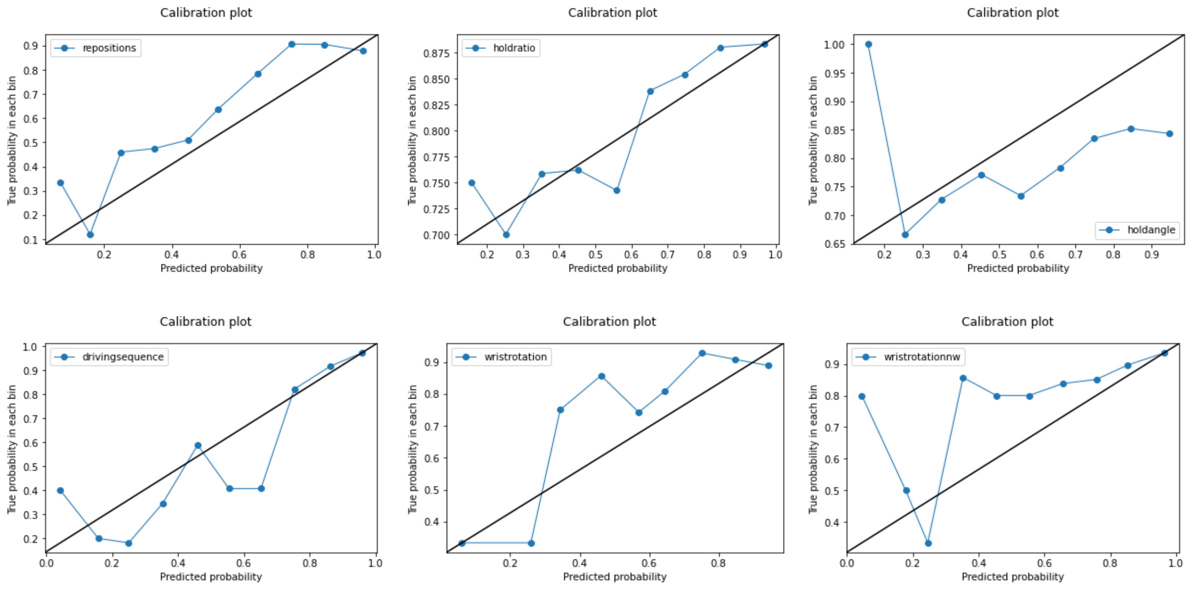

Supplementary Figure 1. Calibration plots for each sub-skill

We study the effectiveness of the prior known relationships. For comparison, we consider the non-informative associations among sub-skills, i.e., the sub-skills are related to each other without any prior bias (equal possibility). We compare the performance of joint skill assessment with the prior known relationships and with the non-informative relationships. Results are shown in Table 1. As shown, the performance of joint skill assessment with non-informative relationships is significantly worse than the performance with prior known relationships. Irrelevant information can be introduced as noise given the non-informative relationships.

Supplementary Table 1. Effectiveness of prior inter-dependencies. Boldfaced denotes best and ± are standard deviations across 5 held-out institutions (AUC).

Sub-skill	w/o Prior Known Relationships	w Prior Known Relationships
Needle Repositioning	0.79±0.03	**0.80±0.03**
Needle Hold Ratio	0.55±0.05	**0.60±0.03**
Needle Hold Angle	0.47±0.10	**0.59±0.04**
Driving Smoothness	**0.88±0.06**	0.86±0.04
Wrist Rotation	0.61±0.05	**0.64±0.06**
Wrist Rotation NW	0.63±0.10	**0.69±0.04**
**MEAN**	0.66±0.03	**0.70±0.02**

Furthermore, we visualize the learned attentions using non-informative relationships among sub-skills in Figure 2 for

comparison. As shown, the learned attention maps from different institutions are quite different. These results show that data-dependent attentions are not generalizable across institutions, further demonstrating the effectiveness of the proposed prior known relationships among sub-skills.

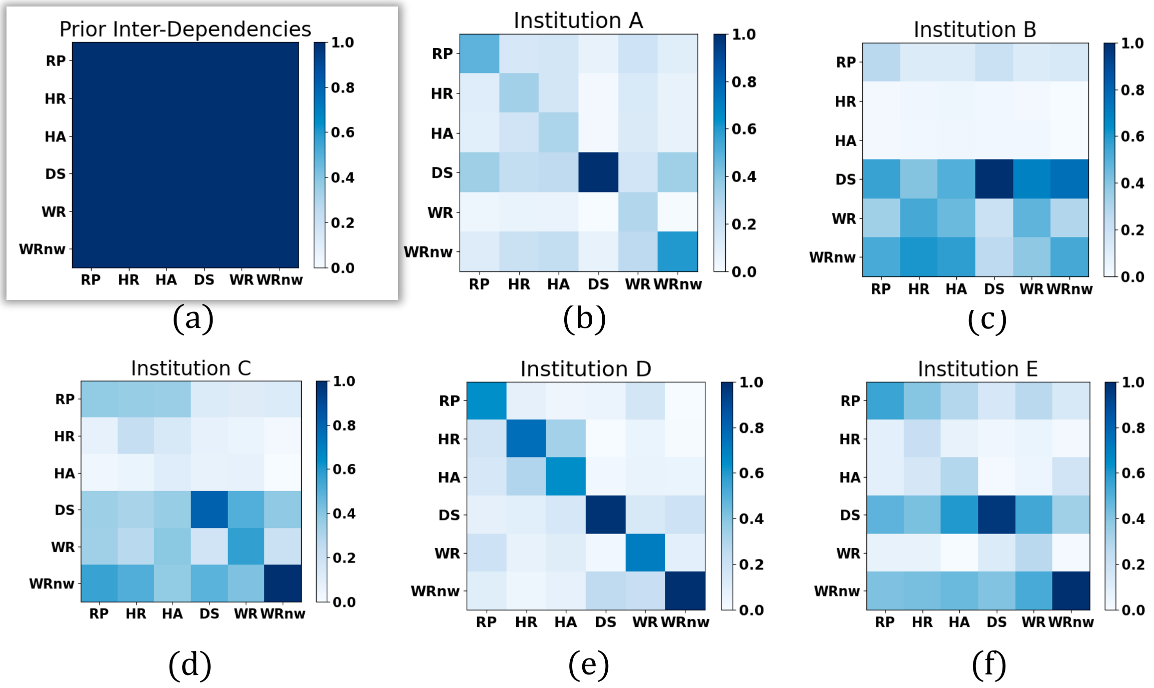

Supplementary Figure 2. Visualization of non-informative equal relationships among sub-skills.
